# Immediate radiographic reduction loss but preserved clinical outcomes after hardware removal in lateral clavicle fractures treated with plate fixation and coracoclavicular augmentation

**DOI:** 10.1186/s12891-026-09727-8

**Published:** 2026-03-17

**Authors:** Malik Jessen, Lennart Gerdesmeyer, Philipp Zehnder, Michael Zyskowski, Peter Biberthaler, Chlodwig Kirchhoff, Markus Schwarz

**Affiliations:** 1 Department of Sports Orthopedics, TUM University Hospital Rechts der Isar, Ismaninger Str. 22, Munich, 81675 Germany; 2 Department of Trauma Surgery, TUM University Hospital Rechts der Isar, Ismaninger Str. 22, Munich, 81675 Germany

**Keywords:** Lateral clavicle fracture, Hardware removal, Coracoclavicular augmentation, Locking plate, Coracoclavicular distance, Tunnel widening, Functional outcome, Patient-reported outcome measures (PROMs)

## Abstract

**Background:**

Locking plate fixation combined with coracoclavicular (CC) augmentation using a suspensory fixation system is a widely accepted technique for unstable lateral clavicle fractures. Elective hardware removal is commonly performed due to mechanical irritation or discomfort, yet data on radiographic and clinical outcomes following implant removal remain limited. The purpose of this study was to evaluate immediate radiographic loss of reduction and long-term clinical outcomes after removal of the locking plate and CC augmentation in patients with lateral clavicle fractures. It was hypothesized that a measurable loss of reduction occurs following hardware removal, and that clinical outcomes would nevertheless remain excellent, with no residual patient complaints.

**Methods:**

A total of 41 patients with lateral clavicle fractures treated with locking plate fixation and CC augmentation underwent implant removal between 2013 and 2022. Coracoclavicular distance (CCD) was measured pre- and immediately post-removal. Clavicular drill tunnel (CDT) diameters were measured post-removal. Clinical outcomes were assessed using PROMs (CMS, DASH, SPADI, SSV, VAS), as well as return to work and sports, and re-operation rates.

**Results:**

The mean CCD increased from 9.1 ± 3.2 mm to 10.0 ± 3.0 mm after implant removal (*p* < .0001). A loss of reduction ≥ 10% was observed in 54% of cases, and a substantial loss (≥ 6 mm) in 12%. CDT measurements showed significant tunnel widening toward the inferior cortex compared to the superior and mid-clavicular cortex (*p* < .0001). Clinically, 23 patients were included after a mean follow-up of 71 months. Patients achieved excellent outcomes with a mean CMS of 88.5 ± 10.5, DASH of 5.4 ± 7.6, SPADI of 93.4 ± 7.9, SSV of 95.4 ± 6.9, and VAS of 0.6 ± 1.1. PROMs did not differ between patients with or without radiographic loss of reduction. All patients returned to work and sports, with no reoperations required.

**Conclusions:**

Although 54% of patients showed a measurable radiographic loss of reduction following implant removal, substantial displacement was uncommon (12%). Radiographic loss of reduction was not associated with inferior clinical outcomes. Patients demonstrated excellent long-term function, with a full return to work and sports.

## Background

Lateral clavicle fractures account for approximately 10% to 30% of all clavicle fractures and are frequently associated with the disruption of the coracoclavicular (CC) ligaments [1–3].

Locking plate fixation combined with CC augmentation using a suspensory fixation system is widely used to restore stability in unstable lateral clavicle fractures, and excellent outcomes have been reported [4–7], particularly in Neer type IIb lateral clavicle fractures with stand-alone CC augmentation [4, 8–11].

Despite reliable fracture healing, many patients request elective hardware removal due to cosmetic concerns, mechanical irritation, or persistent discomfort from the plate or button–suture construct [12–17]. Although implant removal is commonly performed [14], data on radiographic or clinical consequences remain limited [15, 18]. Concerns include potential loss of reduction after removal of CC augmentation, tunnel widening, or secondary instability [15, 19, 20]. Previous studies have primarily investigated tunnel widening or reduction loss following CC augmentation for acromioclavicular (AC) joint injuries [12, 15, 20, 21], yet no study has evaluated these parameters specifically after implant removal in the context of lateral clavicle fractures.

This lack of evidence is clinically relevant because surgeons frequently perform implant removal without being able to counsel patients adequately on potential radiographic changes or functional implications. It also remains unclear whether reduction loss occurs only after implant removal or already develops during the postoperative course.

The purpose of this study was to analyze radiographic and clinical outcomes after hardware removal following locking plate fixation and CC augmentation for lateral clavicle fractures. The primary aims were to assess immediate changes in coracoclavicular distance (CCD) and to determine the rate of loss of reduction. Secondary aims included evaluating the clavicular drill tunnel diameter (CDT), functional outcome scores, and return to work and sports. It was hypothesized that measurable loss of reduction occurs after hardware removal, but that clinical outcomes remain excellent.

## Methods

### Patient selection

Institutional Review Board approval was obtained prior to this retrospective study (Technical University of Munich, 2023-483-S-SB). Reporting followed STROBE guidelines [22]. Patients who underwent elective removal of plate osteosynthesis and high-tensile suture tape CC augmentation between January 2013 and December 2022 were screened. Hardware removal was performed for cosmetic concerns, mechanical irritation, or persistent discomfort unresponsive to conservative treatment.

Inclusion criteria were set as follows: patients aged 18 years or older with an acute lateral clavicle fracture at index surgery (≤ 3 weeks post-injury), treated with primary coracoclavicular (CC) stabilization using a high-tensile suture tape suspensory fixation system (Arthrex, Naples, FL, USA), with radiologically confirmed fracture consolidation before implant removal, and with available pre- and post-removal x-ray imaging in two planes. The pre-removal x-ray had to be obtained within four weeks before hardware removal, and the postoperative radiograph had to be acquired no later than one day after surgery. A minimum clinical follow-up duration of 18 months was required for inclusion in the clinical outcome analysis. Exclusion criteria included pathological fractures, re-fractures at initial surgery, medial or shaft clavicle fractures, associated fractures of the ipsilateral shoulder, incomplete radiographic documentation, and lateral clavicle fractures treated solely with plate osteosynthesis without CC augmentation using the high-tensile suture tape system.

### Surgical technique and implant removal

The index surgery consisted of locking plate fixation (distal clavicle fracture plate, Arthrex, Naples, FL, USA) in combination with arthroscopically assisted coracoclavicular (CC) augmentation using the DogBone^®^ system (Arthrex, Naples, FL, USA). No additional acromioclavicular (AC) cerclage was performed. Implant removal was carried out under general anesthesia through the original surgical approach by two surgeons with specialization in shoulder surgery, ensuring standardized technique and consistency across procedures. The procedure included open removal of the locking plate and the clavicular button of the CC suture-button construct, while the coracoid button was left in situ. No additional arthroscopy was performed during implant removal. The surgical incisions were closed in a sterile manner. Radiographs in two planes (a/p and 20° cephalad) were obtained on the day of surgery or the day after to verify complete removal of hardware.

### Postoperative rehabilitation

Postoperative care included a standardized rehabilitation protocol. Patients were allowed to use a sling for comfort only during the first few days postoperatively. Free range of motion of the shoulder from the first postoperative day was permitted. High-impact sports activities were discouraged for six weeks after surgery. As part of the standardized postoperative protocol, all patients were routinely invited for a clinical follow-up visit at six weeks after hardware removal.

### Radiographic assessment

**Fig. 1:**
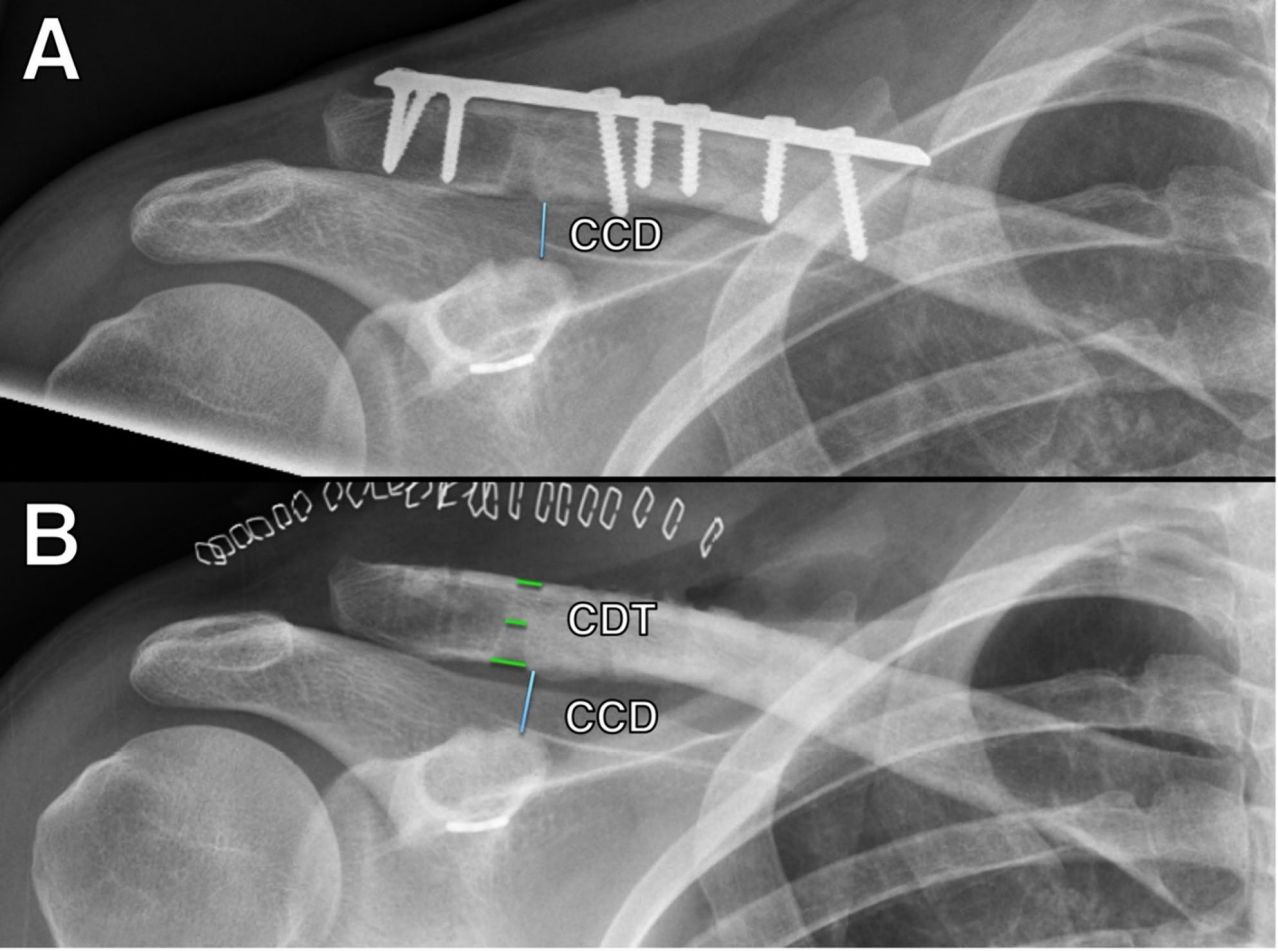
Measurement protocol. **A** Pre-removal radiograph illustrating measurement of the Coracoclavicular Distance (CCD, indicated by blue line), defined as the distance between the coracoid process’s tip and the clavicle’s inferior cortex. **B** Post-removal radiograph illustrating measurement of the CCD and Clavicular Drill Tunnel (CDT, indicated by green lines) diameters at three defined levels: superior cortex, mid-clavicular cortex, and inferior cortex. All measurements were performed on standardized, true anteroposterior radiographs with the beam perpendicular to the scapular plane

All radiographic measurements and parameters were assessed using a picture archiving and communication system (PACS) workstation certified for clinical use (IDS7 21.2, Sectra AB, Linköping, Sweden). Radiographic evaluation was based on pre-removal and immediate post-removal anteroposterior (unweighted) radiographs of the clavicle (Fig. 1). Fractures were classified according to the modified Neer and Cho systems [23, 24]. All cases represented unstable lateral clavicle fractures (modified Neer IIb; Cho IIb or IIC), characterized by involvement of the coracoclavicular ligaments. All radiographs were obtained in a standardized true anteroposterior projection with the beam perpendicular to the scapular plane and no tilt. Radiographs with oblique or non-standard views (e.g., 15° tilt) were excluded to ensure consistency of measurements. The coracoclavicular distance (CCD) was measured as the distance between the tip of the coracoid process and the inferior cortex of the clavicle, as previously described [15, 25, 26]. The CCD was measured on the final radiograph obtained immediately before implant removal and on the first radiograph obtained immediately after removal. Loss of reduction after hardware removal was defined as a ≥ 10% increase in CCD on the post-removal radiograph compared with the pre-removal radiograph. Substantial loss was defined as an absolute increase of ≥ 6 mm post-removal, consistent with previously proposed thresholds [15, 27–29]. Horizontal instability was not assessed, as stress radiographs in cross-body adduction views were not routinely performed immediately after surgery. Clavicular drill tunnel (CDT) diameters were measured at the superior, middle, and inferior cortices on the same radiographs as previously described [12, 21].

### Clinical assessment

One examiner (M.J.), who was not involved in the surgical treatment, evaluated patient outcomes via mail and telephone contact using a standardized questionnaire. The assessment included the Munich Shoulder Questionnaire (MSQ), which integrates the Constant-Murley Score (CMS), the Disabilities of the Arm, Shoulder, and Hand (DASH) score, and the Shoulder Pain and Disability Index (SPADI) [30]. The Subjective Shoulder Value (SSV) was also recorded. Pain levels were assessed using a visual analogue scale (VAS). Additionally, patients were asked about their return to sports, work, and any subsequent medical consultations following the removal of the hardware.

### Statistical analysis

Two independent raters (M.J., L.G.) performed all measurements at different time points. Interrater and intrarater reliability were assessed using the intraclass correlation coefficient (ICC) for all measurements, with 95% confidence intervals. Intrarater reliability was evaluated by a single rater (M.J.), with repeated measurements taken at least six weeks apart. Agreement levels were interpreted according to the guidelines by Landis and Koch [31], which define ICC values as follows: 0.00–0.20 as slight agreement, 0.21–0.40 as fair agreement, 0.41–0.60 as moderate agreement, 0.61–0.80 as substantial agreement, and 0.81–1.00 as strong agreement. The Kolmogorov-Smirnov test was used to assess normal distribution. The paired t-test (for parametric distributions) or the Mann-Whitney test (for nonparametric distributions) was used to compare continuous variables within patients. Categorical variables (sex, fracture side, and the proportion of patients with any or substantial loss of reduction) were compared using Fisher’s exact test due to small sample sizes and expected cell counts. Results for parametric data are reported as means with standard deviations. Two-tailed P values were calculated, with statistical significance set at *P* < .05. Data analysis was performed using GraphPad Prism for macOS, Version 10.0.3 (GraphPad Software, San Diego, California, USA) and IBM SPSS Statistics for macOS, Version 30.0.0.0 (IBM Corp., Armonk, New York, USA).

## Results

**Fig. 2:**
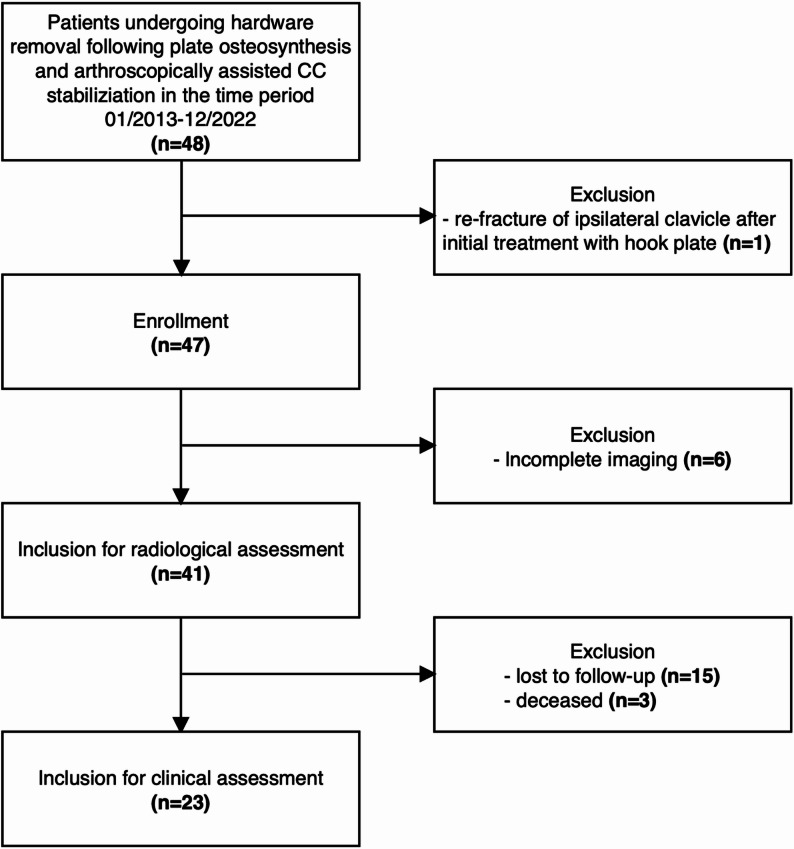
Flow chart illustrating the patient selection process and final inclusion for radiological and clinical assessment. The chart details the reasons for exclusion at each stage of the study

A total of 48 patients underwent hardware removal after locking plate fixation with arthroscopically assisted CC augmentation between 01/2013 and 12/2022. Seven patients were excluded (re-fracture, *n* = 1; incomplete imaging, *n* = 6), leaving 41 patients for radiographic analysis. Fifteen patients were lost to follow-up, and three had died; thus, 23 patients (follow-up rate of 60.5%) were available for clinical assessment (Fig. 2; Table 1).

**Table 1:** Baseline characteristics of the overall study cohort and the subgroup with clinical follow-up. SD = Standard deviation; CCD = Coracoclavicular distance; CDT = Clavicular drill tunnel; ORIF = Open reduction and internal fixation

Variable		Radiological Assessment	Clinical Assessment
Patients		41	23
Sex			
	Male	24 (59%)	16 (70%)
	Female	17 (41%)	7 (30%)
Side			
	Right	14 (34%)	12 (52%)
	Left	27 (66%)	11 (48%)
Age at hardware removal in years [SD]		44 [13]	45 [15]
Mean time between ORIF and implant removal in months [SD]			
		18 [8]	16 [7]
Mean Follow-Up in months [SD]			71 [38]

### Radiographic analysis

**Fig. 3:**
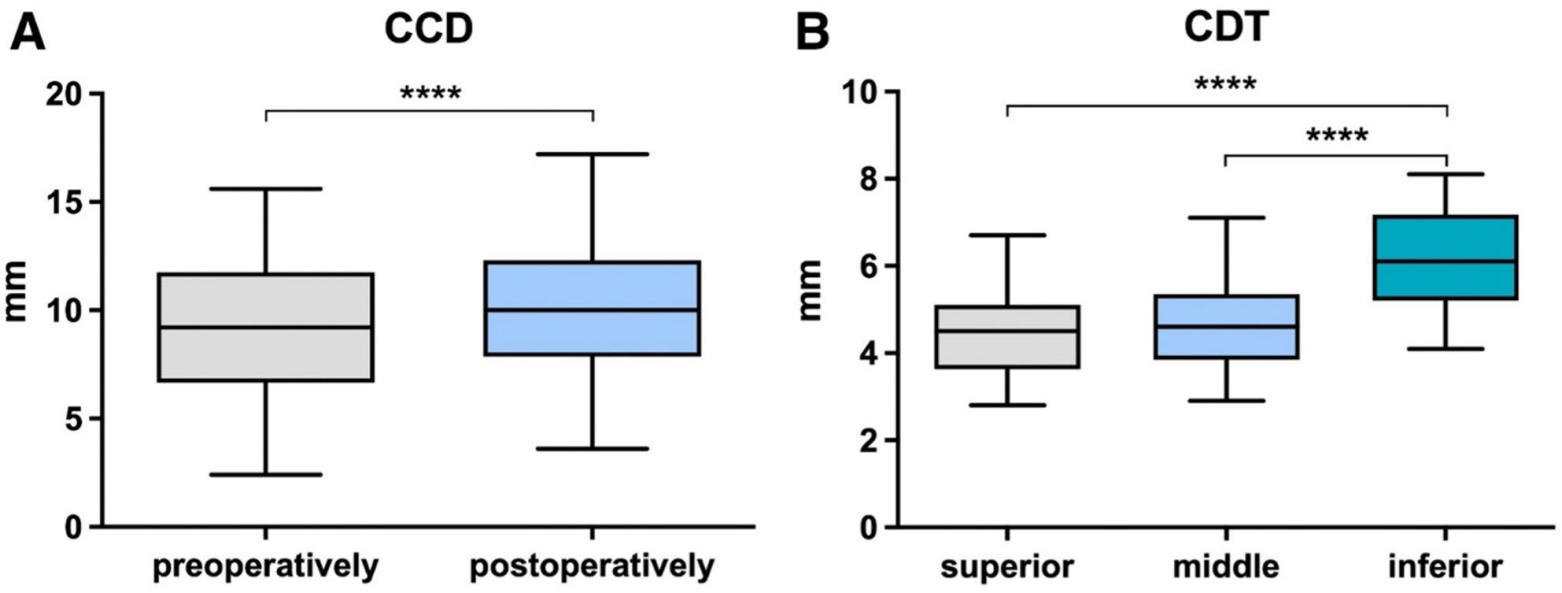
Box plots demonstrating (**A**) Coracoclavicular distance (CCD) measurements preoperatively and postoperatively, and (**B**) Clavicular drill tunnel (CDT) diameter at the superior, middle, and inferior levels. Boxes represent median values with Interquartile ranges (IQR), and whiskers indicate minimum and maximum values. A significant increase in CCD was observed postoperatively (****P < .0001). CDT measurements revealed substantial differences between the superior, middle, and inferior levels (****P < .0001)

The mean CCD increased from 9.1 ± 3.2 mm pre-removal to 10.0 ± 3.0 mm post-removal (Fig. 3, *p* < .0001). A loss of reduction ≥ 10% occurred in 54% (22/41) of patients, and a substantial loss (≥ 6 mm) in 12% (5/41).

Post-removal CDT diameters were 4.5 ± 1.0 mm (superior), 4.7 ± 1.0 mm (middle), and 6.1 ± 1.1 mm (inferior), with significant differences between levels (*p* < .0001).

To assess potential selection bias, baseline characteristics and radiographic parameters were compared between patients with and without clinical follow-up (Table 2). No significant differences were observed for demographic variables or radiographic parameters, whereas fracture side distribution differed between groups (*p* < .01).

**Table 2:** Baseline characteristics and radiographic parameters of patients with only radiological assessment and patients with both radiological and clinical assessment. SD = Standard deviation; CCD = Coracoclavicular distance; CDT = Clavicular drill tunnel; ORIF = Open reduction and internal fixation

Variable		Only Radiological Assessment	Radiological and Clinical Assessment	*p*-value
Patients		18	23	
Sex				
	Male	8 (44%)	16 (70%)	0.12
	Female	10 (56%)	7 (30%)	
Side				
	Right	2 (11%)	12 (52%)	< 0.01
	Left	16 (89%)	11 (48%)	
Age at hardware removal in years [SD]		43 [12]	45 [15]	0.71
Mean time between ORIF and implant removal in months [SD]				
		20 [9]	16 [7]	0.20
Mean CCD before implant removal in mm [SD]		8.6 [3.7]	9.5 [2.8]	0.40
Mean CCD after implant removal in mm [SD]		9.6 [3.9]	10.4 [2.3]	0.44
Number of patients with loss of reduction ≥ 10%		10	12	1.0
Number of patients with substantial loss (≥ 6 mm)		3	2	0.64
Mean CDT superior in mm [SD]		4.6 [1.0]	4.4 [1.0]	0.75
Mean CDT middle in mm [SD]		4.7 [1.1]	4.6 [1.0]	0.64
Mean CDT inferior in mm [SD]		5.8 [1.1]	6.4 [1.1]	0.16

### Clinical outcomes

Twenty-three patients completed the clinical assessment after a mean follow-up of 70.7 months (± 37.1, range: 18 to 128 months). Indications for implant removal were mechanical irritation or discomfort (*n* = 17), pain (*n* = 3), restricted range of motion (*n* = 2), and cosmetic concerns (*n* = 1).

Functional results were excellent: mean CMS 88.5 ± 10.5, DASH 5.4 ± 7.6, SPADI 93.4 ± 7.9, SSV 95.4 ± 6.9, and VAS 0.6 ± 1.1.

Patient-reported outcomes did not differ between patients with (*n* = 12) and without (*n* = 11) loss of reduction. Mean CMS were 88.7 ± 12.2 points in patients without loss and 88.3 ± 9.2 points in those with loss (*p* = .80). SSV values were 94.1 ± 8.6% versus 96.6 ± 5.0% (*p* = .51), SPADI scores 92.6 ± 9.7 versus 94.2 ± 6.2 (*p* = .88), and DASH scores 6.2 ± 8.1 versus 4.2 ± 7.1 (*p* = .29). VAS pain levels were similarly low in both groups (0.6 ± 1.3 versus 0.7 ± 0.8; *p* = .46). All observed group differences in PROMs were below established minimal clinically important difference (MCID) thresholds [32–37].

All patients returned to work; the mean time to any occupational reintegration was 1.2 ± 1.1 weeks, and full capacity was reached after 1.8 ± 2.0 weeks. Six patients (26%) reported an improvement in occupational performance after hardware removal.

Of the 23 patients who completed the clinical assessment, 22 (96%) reported regular participation in sporting activites, while one patient (4%) stated being non-active. The most common activity category was endurance/low-impact sports (17 patients, 74%), followed by strength and fitness training (10 patients, 43%), overhead and racket sports (6 patients, 26%), winter sports such as skiing or snowboarding (5 patients, 22%), and aquatic sports including swimming or surfing (5 patients, 22%). All patients with preinjury sports participation (*n* = 22) returned to sports postoperatively.

Resumption of sporting activity occurred after 2.6 ± 2.2 weeks, and full return to pre-injury level after 4.7 ± 3.9 weeks. Thirteen patients (57%) reported an improvement in their sporting performance; no patient reported deterioration.

### Interrater and intrarater reliability

Interrater reliability was strong for all measurements, with ICCs of 0.988 (95% CI, 0.978–0.994) for pre-removal CCD, 0.990 (95% CI, 0.982–0.995) for post-removal CCD, 0.929 (95% CI, 0.846–0.967) for CDT at the superior level, 0.945 (95% CI, 0.882–0.975) for CDT at the middle level, and 0.863 (95% CI, 0.703–0.936) for CDT at the inferior level.

Intrarater reliability was similarly strong, with ICCs of 0.996 (95% CI, 0.992–0.998) for pre-removal CCD, 0.996 (95% CI, 0.993–0.998) for post-removal CCD, 0.968 (95% CI, 0.930–0.985) for CDT at the superior level, 0.967 (95% CI, 0.929–0.985) for CDT at the middle level, and 0.913 (95% CI, 0.812–0.960) for CDT at the inferior level.

## Discussion

The principal finding of this study is that hardware removal after locking plate fixation and coracoclavicular (CC) augmentation for lateral clavicle fractures resulted in a radiographic loss of reduction in 54% and a substantial loss in 12% of cases. Despite this, functional outcomes remained excellent after a mean follow-up period of 71 months, with all patients returning to work and resuming their participation in sports.

The radiological findings are consistent with previous investigations of loss of reduction after hardware removal [15]. Rupp et al. evaluated 22 patients following implant removal after CC augmentation for AC joint dislocation and observed an immediate radiographic increase in coracoclavicular distance from 12.6 ± 3.7 mm to 14.5 ± 3.3 mm. In their cohort, 10 patients (45%) demonstrated a measurable loss of reduction, with substantial loss occurring in one patient (4.5%). In the present radiographic analysis of 41 patients, a comparable rate of immediate loss of reduction was observed in 54% of cases, while substantial loss was more frequent, occurring in 12% of cases.

Both the data presented by Rupp et al. and the findings of the present study demonstrate that an immediate loss of reduction after hardware removal following CC augmentation is a consistent observation, regardless of whether the initial procedure was performed for an AC joint dislocation or a lateral clavicle fracture stabilized with additional plate osteosynthesis.

A progressive loss of reduction over time has also been described following CC augmentation for AC joint dislocation [19, 20]. However, neither the current study nor the study by Rupp et al. included radiographic data from the time of initial surgery [15].

Some degree of loss of reduction may occur between the index procedure and implant removal. However, the most pronounced change is expected at the moment of hardware removal, when the mechanical support of the fixation device is lost. During the preceding healing phase, reduction typically remains stable, supported by graft maturation and implant fixation. Accordingly, the postoperative radiograph obtained immediately after implant removal represents the primary point at which vertical displacement may reappear.

Notably, the present data demonstrate a slightly higher rate of substantial loss (≥ 6 mm), which may be explained by the additional mechanical unloading that occurs with the removal of both the locking plate and the clavicular button in lateral clavicle fractures, as opposed to isolated CC fixation in AC joint injuries. In contrast, the AC joint stabilization cohort analyzed by Rupp et al. routinely included an additional AC cerclage, which likely provided supplementary stability in both horizontal and rotational directions. This adjunct is not typically applied in fracture fixation, which may account for the slightly greater loss of reduction observed in the present study.

Whether this secondary loss of reduction after hardware removal has any clinical relevance, however, has not yet been investigated in the existing literature. In the present cohort, radiographic loss of reduction was common, yet it showed no association with patient-reported outcomes. CMS, SSV, SPADI, DASH, and VAS were comparable between patients with and without loss of reduction, with no significant differences across any domain. These findings suggest that a moderate postoperative increase in CCD may not necessarily influence clinical outcomes.

Regarding the CDT diameters, significant tunnel widening toward the inferior cortex was observed, consistent with earlier findings in both acute and chronic stabilization procedures following AC joint dislocation. Berthold et al. and Bellmann et al. have demonstrated that inferior tunnel expansion is correlated with biomechanical micromotion and may predispose to reduction loss or failure in revision settings [12, 25]. A clinical study has also shown a positive correlation between tunnel widening and coracoclavicular loss of reduction [38]. Tunnel widening occurs in up to 70% of cases following CC augmentation using suspensory stabilization techniques [39].

Although tunnel widening, particularly at the inferior cortex, was evident on postoperative imaging, the present data do not allow definitive conclusions regarding its functional relevance. The study was not specifically designed to detect associations between radiographic tunnel morphology and postoperative shoulder function, as no dedicated correlation analyses or dynamic stability assessments were performed. Accordingly, tunnel widening should be interpreted as a radiographic observation rather than a validated surrogate of clinical or biomechanical instability.

Clinically, patients demonstrated excellent functional outcomes, with high CMS, SPADI, and SSV scores and low DASH and VAS scores at a mean follow-up of 71 months after hardware removal. Furthermore, 100% of patients in the presented cohort returned to work and sports, underscoring the long-term functional success of this approach, even following implant removal. Thus, no apparent adverse clinical effect of a loss of reduction following hardware removal can be identified.

This disconnect between imaging findings and patient-reported outcomes underscores the limited predictive value of static radiographic parameters for functional recovery in patients who underwent combined fixation and implant removal.

To our knowledge, no previous studies have reported clinical follow-up data after hardware removal following CC augmentation, making these findings the first in the current literature.

Another aspect of the presented patient cohort was that no additional arthroscopy was performed to retrieve the coracoid button. Although some centers routinely remove both ends of the suspensory fixation device [15], evidence supporting the clinical necessity of arthroscopic button retrieval is still lacking. In the current series, none of the patients who retained the coracoid button reported coracoid-region pain, symptoms suggestive of subcoracoid impingement, or mechanical irritation at long-term follow-up. These observations indicate that button retention did not result in any apparent clinical disadvantage in this cohort. However, these findings should be interpreted cautiously. The study was not designed or powered to evaluate the potential risks or benefits of coracoid button retention, nor did it include imaging specifically assessing the subcoracoid space or possible osteolysis. Consequently, the absence of symptoms in this cohort should be considered hypothesis-generating rather than practice-changing.

Routine arthroscopic retrieval of the coracoid button cannot be recommended or dismissed based on the present data. Future comparative studies with dedicated imaging and larger sample sizes are needed to determine whether coracoid button retention is truly safe and clinically equivalent to removal.

From a clinical perspective, the study’s findings offer meaningful implications for surgical decision-making and patient counseling. Patients frequently request elective hardware removal due to irritation or psychological discomfort; however, surgeons have historically lacked data to predict the structural or functional consequences of this procedure. The present results provide reassurance that minor radiographic changes after implant removal are not associated with a decline in shoulder function or patient satisfaction.

This enables a more informed and confident approach to shared decision-making, allowing clinicians to explain that while measurable radiographic changes may occur, they are not clinically detrimental.

Despite the strengths of this study—including standardized radiographic measurement, long-term follow-up, and validated PROMs—some limitations must be acknowledged. The follow-up rate for clinical assessment was 60.5%, which may introduce selection bias. Stress radiographs were not performed, and horizontal instability was not assessed. The only baseline difference between patients with and without clinical follow-up was the distribution of fracture sides (Table 2). Since the side was not related to CCD or CDT values and did not affect the rates of loss of reduction, this imbalance is unlikely to represent a relevant source of selection bias.

The timing of radiographic assessment requires careful interpretation. CCD measurements were obtained within four weeks prior to implant removal and again immediately after removal. Although this design cannot fully disentangle gradual postoperative settling from the immediate biomechanical effect of removing the fixation device, the likelihood of relevant cumulative changes in the final pre-removal interval is low. The mean time between the index procedure and hardware removal was 18 ± 8 months, a stage at which fracture healing, graft maturation, and biological integration of the augmentation construct are typically complete. Consequently, substantial changes in clavicular position within the last few weeks before implant removal are improbable.

In this context, the observed postoperative increase in CCD should be interpreted as the net effect resulting from the combination of the pre-removal state and the immediate unloading after removal of the fixation device, without implying direct causality. This clarification has been incorporated into the Discussion, and the Conclusions have been adjusted to avoid overstating causative interpretation.

The present study assessed only vertical radiographic stability using CCD measurements and did not evaluate horizontal translation. Clinically, neither vertical nor horizontal instability nor scapular dyskinesis was examined, and no dynamic or stress imaging was performed. As a result, subtle multidirectional or dynamic instability cannot be ruled out. Although PROMs in the clinical follow-up subgroup did not indicate clinically meaningful impairment, these outcomes alone may not detect fine abnormalities in shoulder girdle mechanics, particularly in overhead athletes or manual laborers. This limitation should be considered when interpreting the postoperative stability profile.

Furthermore, the findings of this study apply to a highly standardized construct, consisting of a locking plate fixation combined with DogBone^®^ CC augmentation and a retained coracoid button, without additional AC cerclage. As implant design, graft configuration, and removal strategies vary across techniques, the results should not be directly extrapolated to other CC devices or constructs. Further studies comparing different augmentation systems are required to determine whether these observations hold true in other settings.

Future research should investigate whether specific patient subgroups (e.g., overhead athletes, manual laborers) are more susceptible to subtle impairments from minor loss of reduction, and whether dynamic imaging or functional testing can reveal additional deficits not captured by standard PROMs.

Additionally, prospective studies are needed to evaluate long-term outcomes in patients with marked tunnel widening or residual instability, especially in the context of potential revision surgery.

## Conclusions

Following hardware removal after locking plate fixation and coracoclavicular augmentation for lateral clavicle fractures, radiographic loss of reduction was observed in more than half of the patients, whereas substantial loss was uncommon. These radiographic changes did not translate into measurable clinical impairment. Patients with and without radiographic loss of reduction demonstrated comparable PROMs at long-term follow-up, with consistently high functional scores and reliable return to work and sports.

## Data Availability

The datasets generated and analysed during the current study are available from the corresponding author on reasonable request.

## Abbreviations

AC: Acromioclavicular
ACCR: Anatomic Coracoclavicular Ligament Reconstruction
AP: Anteroposterior
CC: Coracoclavicular
CCD: Coracoclavicular Distance
CDT: Clavicular Drill Tunnel
CMS: Constant–Murley Score
DASH: Disabilities of the Arm, Shoulder, and Hand
ICC: Intraclass Correlation Coefficient
IQR: Interquartile Range
MSQ: Munich Shoulder Questionnaire
ORIF: Open Reduction and Internal Fixation
PACS: Picture Archiving and Communication System
PROMs: Patient–Reported Outcome Measures
SD: Standard Deviation
SPADI: Shoulder Pain and Disability Index
SSV: Subjective Shoulder Value
STROBE: Strengthening the Reporting of Observational Studies in Epidemiology
VAS: Visual Analogue Scale
